# Efficacy and safety associated with immune checkpoint inhibitors in unresectable hepatocellular carcinoma: A re‐analysis of a meta‐analysis

**DOI:** 10.1002/jgh3.12856

**Published:** 2023-01-24

**Authors:** Shouhao Zhou, Chan Shen

**Affiliations:** ^1^ Department of Public Health Sciences, College of Medicine Pennsylvania State University Hershey Pennsylvania USA; ^2^ Penn State Cancer Institute Pennsylvania State University Hershey Pennsylvania USA; ^3^ Department of Surgery, College of Medicine Pennsylvania State University Hershey Pennsylvania USA

**Keywords:** Bayesian statistics, immune checkpoint inhibitors, meta‐analysis, predictive intervals, unresectable hepatocellular carcinoma

## Abstract

We identified analytic limitations in a recent meta‐analysis and re‐examined the efficacy and safety associated with immune checkpoint inhibitors (ICIs) in unresectable hepatocellular carcinoma (HCC) compared with standard therapies. Our findings mostly contradict conclusions from the previous study, suggesting the need for continuing the investigation of ICIs in HCC with additional clinical evidence.

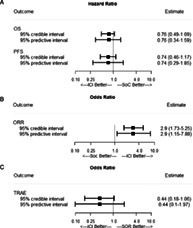

## Introduction

The systematic review by Jácome *et al*. examined the efficacy and safety associated with immune checkpoint inhibitors (ICIs) in patients with unresectable hepatocellular carcinoma (HCC).^1^ Specifically, they documented a literature search and selected three randomized clinical trials eligible for meta‐analysis. From a statistical perspective, data analysis should be conducted with caution given the very small number of studies.^2^, ^3^ In the presence of heterogeneity and few included studies, conventional likelihood‐based frequentist methods fail to reach the nominal level of coverage probability for the overall effect, resulting in an inflated type‐I error in meta‐analyses.^4^ In contrast, Bayesian modeling, with a sensible prior, can overcome this limitation by naturally capturing heterogeneity between studies and delivering exact inference for decent coverage probability estimation.^4^

## Methods

To derive the statistical inference with proper uncertainty quantification, we applied a Bayesian model using half‐normal prior with a scale of 0.5 for the between‐study heterogeneity^5^ and estimated the hazard ratios (HRs) for survival outcomes and odds ratio (OR) for event incidences with 95% credible intervals (CrI) and 95% predictive intervals (PrI).^6^ The joint posterior distributions of model parameters were generated using Markov chain Monte Carlo simulation and implemented in JAGS 4.3.0. Sensitivity analyses were conducted to examine the impact of various prior distributions using alternative approaches.

## Results

**Figure 1:**
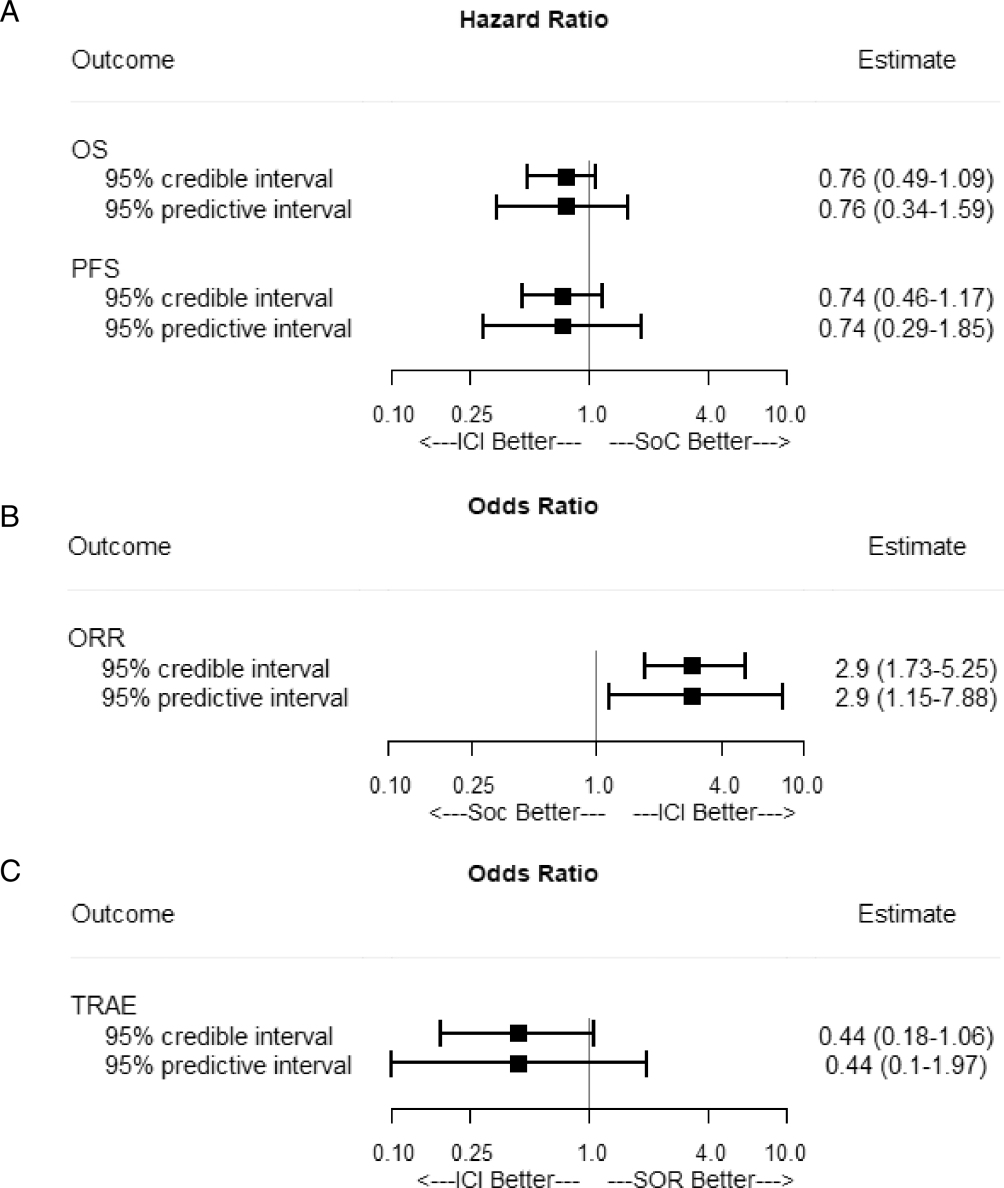
Meta‐analytic re‐assessment of efficacy and safety associated with immune checkpoint inhibitors (ICIs) in patients with unresectable hepatocellular carcinoma (HCC). Each square shows the posterior median of hazard ratio for overall survival (OS) and progression‐free survival (PFS) or of odds ratio for overall response rate (ORR) and treatment‐related adverse events (TRAEs). The horizontal bands show the corresponding 95% credible intervals or 95% predictive intervals. The control group in comparisons of (a) and (b) was standard of care (SoC), which includes placebo or sorafenib (SOR). The control group in comparison of (c) was sorafenib.

Compared with standard therapies, ICIs were only associated with a significantly improved overall response rate (OR, 2.9; 95% CrI, 1.73–5.25; 95% PrI, 1.15–7.88, Fig. 1b). However, the improvement of ICIs in both overall survival (HR, 0.76; 95% CrI, 0.49–1.09; 95% PrI, 0.34–1.59) and progression‐free survival (HR, 0.74; 95% CrI, 0.46–1.17; 95% PrI, 0.29–1.85) was no longer significant (Fig. 1a); nor was the probability of grade 3 or 4 treatment‐related adverse events significanly lower with ICIs than with sorafenib (OR, 0.44; 95% CrI, 0.18–1.06; 95% PrI, 0.1–1.97, Fig. 1c). In sensitivity analysis, even wider Bayesian credible intervals were derived using either half‐normal prior with scale 1 or half‐Cauchy prior with scale 10 for the between‐study heterogeneity.

## Discussion

Our re‐analysis of the data shows a substantial difference in results from those of the original publication.^1^ It suggests the critical need of applying appropriate methods in meta‐analysis with few studies and emphasizes the importance of multidisciplinary research, including close collaborations with biostatisticians.

Clinically, it may be too early to conclude that “ICIs should be the new standard of care in systemic therapy of unresectable HCC”, given the crucial limitation of the meta‐analysis with only three eligible studies and the large between‐study heterogeneity. The predictive interval, which also takes into account the between‐study variation in overall uncertainty quantification, can be exploited in sample size determination and power calculation for the planning of future HCC ICI studies.

## References

[1] Jácome AA , Castro ACG , Vasconcelos JPS *et al*. Efficacy and safety associated with immune checkpoint inhibitors in unresectable hepatocellular carcinoma: a meta‐analysis. JAMA Netw. Open. 2021; 4: e2136128. 34870682 10.1001/jamanetworkopen.2021.36128 PMC8649834

[2] Sidik K , Jonkman J . A simple confidence interval for meta‐analysis. Stat. Med. 2002; 21: 3153–9. 12375296 10.1002/sim.1262

[3] Knapp G , Hartung J . Improved tests for a random effects meta‐regression with a single covariate. Stat. Med. 2003; 22: 2693–710. 12939780 10.1002/sim.1482

[4] Seide SE , Röver C , Friede T . Likelihood‐based random‐effects meta‐analysis with few studies: Empirical and simulation studies. BMC Med. Res. Methodol. 2019; 19: 1–14. 30634920 10.1186/s12874-018-0618-3 PMC6330405

[5] Friede T , Röver C , Wandel S , Neuenschwander B . Meta‐analysis of two studies in the presence of heterogeneity with applications in rare diseases. Biom. J. 2017; 59: 658–71. 27754556 10.1002/bimj.201500236 PMC5516158

[6] Higgins JPT , Thompson SG , Spiegelhalter DJ . A re‐evaluation of random‐effects meta‐analysis. J. R Stat. Soc. Ser. A. 2009; 172: 137–59. 10.1111/j.1467-985X.2008.00552.x PMC2667312 19381330

